# Tailoring Household Disaster Preparedness Interventions to Reduce Health Disparities: Nursing Implications from Machine Learning Importance Features from the 2018–2020 FEMA National Household Survey

**DOI:** 10.3390/ijerph21050521

**Published:** 2024-04-23

**Authors:** Meghna Shukla, Taryn Amberson, Tara Heagele, Charleen McNeill, Lavonne Adams, Kevin Ndayishimiye, Jessica Castner

**Affiliations:** 1 College of Nursing, Wayne State University, 5557 Cass Ave, Detroit, MI 48202, USA; av6363@wayne.edu; 2 Castner Incorporated, 1879 Whitehaven Road #150, Grand Island, NY 14072, USAjcastner@albany.edu (J.C.); 3 Health Systems and Population Health School of Public Health, Department of Health Services Research, University of Washington, 1959 NE Pacific St., Seattle, WA 98195, USA; 4 Administration for Strategic Preparedness and Response, National Disaster Medical System, 200 Independence Ave., Washington, DC 20201, USA; 5 Hunter-Bellevue School of Nursing, Hunter College, The City University of New York, 425 East 25th Street, Office 427W, New York, NY 10010, USA; th1591@hunter.cuny.edu; 6 College of Nursing, University of Tennessee Health Science Center’s, Suite 140C, 874 Union Ave., Memphis, TN 38163, USA; cmcneil8@uthsc.edu; 7 Harris College of Nursing & Health Sciences, Texas Christian University, TCU Box 298620, Fort Worth, TX 76129, USA; l.adams2@tcu.edu; 8 Health Policy, Management and Behavior, School of Public Health, University at Albany, 1400 Washington Avenue, Albany, NY 14222, USA

**Keywords:** disasters, disaster preparedness, machine learning, health disparities

## Abstract

Tailored disaster preparedness interventions may be more effective and equitable, yet little is known about specific factors associated with disaster household preparedness for older adults and/or those with African American/Black identities. This study aims to ascertain differences in the importance features of machine learning models of household disaster preparedness for four groups to inform culturally tailored intervention recommendations for nursing practice. A machine learning model was developed and tested by combining data from the 2018, 2019, and 2020 *Federal Emergency Management Agency National Household Survey*. The primary outcome variable was a composite readiness score. A total of 252 variables from 15,048 participants were included. Over 10% of the sample self-identified as African American/Black and 30.3% reported being 65 years of age or older. Importance features varied regarding financial and insurance preparedness, information seeking and transportation between groups. These results reiterate the need for targeted interventions to support financial resilience and equitable resource access. Notably, older adults with Black racial identities were the only group where TV, TV news, and the Weather Channel was a priority feature for household disaster preparedness. Additionally, reliance on public transportation was most important among older adults with Black racial identities, highlighting priority needs for equity in disaster preparedness and policy.

## 1. Introduction

Disasters, both natural and technological (human-caused), continue to pose significant threats to communities across the United States, emphasizing the critical need for effective disaster preparedness strategies. A household can be considered prepared for a disaster if they have developed evacuation and emergency communication plans and set aside a kit of basic supplies needed to endure disaster conditions, such as living without electricity, potable water, and being unable to gain additional supplies for a few days [1,2,3]. Disaster preparedness interventions designed with a one-size-fits-all approach often fail to consider the unique cultural, social, historical, and economic contexts that influence individual/community responses to disasters. Here, we address the need for tailored household disaster preparedness assessments and interventions. 

In a systematic review and meta-analysis of social support, education, and behavior modification interventions for household disaster preparedness, we found a substantial gap in health equity evidence by age, place of residence, race, occupation, gender/sex, religion, education, socioeconomic status, and social capital [4]. For example, Gillum and colleagues [5] and Gielen and colleagues [6] studied household preparedness interventions among participants who predominantly identified as Black or African American (83% and >90%, respectively) with no analysis of racial disparities. For intimate partner violence safety planning at the household level, McFarlane and colleagues [7] demonstrated disparity by age, but not racial identity. In an Eisenman and colleagues [8] study, participants included those with African American identities. However, differences in household disaster preparedness by racial identity were not analyzed. A scoping review on the importance of the ‘social’ in risk narratives of emergency disaster management, preparedness and planning found a lack of engagement when reviewing disaster research with groups covered under the equity, diversity, and inclusion (EDI) umbrella [9]. Additional studies have demonstrated racial and ethnic identity group differences in household preparedness, many have not included geographies where Black/African American populations are included or analyzed [10,11]. Here, we address the household disaster preparedness gap in the evidence on equity interventions with a focus on age and Black/African American racial identity. 

Additional literature review of disaster disparities supports our health disparity focus on older age and Black/African racial identity. Older adults experience disparities in disaster morbidity and mortality as disproportionately low-income [12], socially isolated [13,14,15,16], and victim to disaster crime [17,18,19,20]. Over 70% of older adults in the U.S. have multiple chronic health conditions [21]. While community-dwelling older adults demonstrate higher overall household preparedness, this preparedness still falls short of mitigating disaster morbidity and quality of life risks when considering the care needs for chronic health conditions [12,22,23,24]. When access is diminished to routine chronic disease healthcare during disasters, older adult outcomes are disproportionately affected due to their increased burden of disease [17,25]. For example, older adults comprised the majority of hurricane disaster deaths and hospitalizations for dehydration in Hurricane Sandy, with more traumatic injuries related to household clean-up and foodborne and waterborne disease compared to younger counterparts [26,27,28,29]. Further, households in historically redlined and predominantly Black/African American-identifying urban communities experience macro-level disparities in toxic exposures, post-disaster housing recovery, and community resource access [30,31,32,33].

At-risk populations, including older adults and those from minoritized racial/ethnic groups, bear a disproportionate burden of the adverse impacts from disasters [34]. For example, over half of individuals who died immediately after Hurricane Katrina hit the U.S. in 2005 were over 75 years old [35]. Minoritized communities, particularly African American or Black populations, face systemic disparities that can impact their disaster resilience and preparedness [9,36]. While machine learning (ML) and artificial intelligence (AI) tools may support tailored disaster preparedness assessments and interventions to mitigate health disparities, these tools require careful ethical and equity considerations.

ML- and AI-powered tools remain relatively nascent in household disaster preparedness research. In a population analysis of COVID-19 deaths, Grekousis and colleagues [37] found that older age and African heritage identity were among the top 15 importance features of their machine learning predictive models. Caution is warranted when integrating racial variables in health-related applications. Race-based algorithms can introduce bias in healthcare practices [38]. These algorithms may cause harm by exacerbating bias and disparity, rather than enhancing fairness and objectivity [38,39]. For example, among Black individuals in the U.S., race-based algorithms for kidney health have resulted in falsely elevated estimates of kidney function, contributing to delayed recognition and treatment of kidney disease. Lillywhite and Wolbring [9] found gaps in disaster management, preparedness, and planning technology research where the risk of technology for marginalized groups included in EDI discourse was largely unrealized and only discussed in a positive manner (vs. potential harm), but rarely in conjunction with risk discourse. This indicates a critical, timely gap and an opportunity for disaster research to include questions relevant to risk and marginalized groups. Thus, we address both aggregated and disaggregated analyses with an examination of opportunities to inform nurses in public health and healthcare on promising assessments and interventions to address disaster disparity. 

Nurses, as the largest proportion of the health workforce, are in a key position to provide health education, behavior change interventions, and design social support recommendations to improve disaster preparedness outcomes [4]. The need to bolster disaster education and training across all phases of the disaster management cycle (preparedness, response, recovery, and mitigation) for nurses across work settings remains, as does the need to prepare nurses to prevent and address disaster disparities across the disaster management cycle [40,41,42]. In addition to their more common roles during disasters (i.e., community engagement, patient education/health promotion, first aid, resource allocation, organizational logistics and policy, advanced clinical care, etc.), nurses are well positioned for leadership in disaster management and to ensure at-risk populations are represented in disaster planning, interventions, policy, and response at individual, organizational, community, and societal levels [42,43].

Tailoring public health nursing interventions is associated with improved patient outcomes [44]. Common interventions include population health leadership and disaster program planning; health education on disaster preparedness; direct provision of disaster preparedness assistance care, connection, and referral to additional services or resources; and case management for patients with complex health conditions [45,46,47]. As the field of disaster preparedness evolves, integrating advanced ML/AI technologies into research holds promise for identifying key factors that influence household disaster preparedness and for tailoring interventions accordingly. However, when humans cannot understand how variables (i.e., demographic factors like sex/gender, racial identity, or income) are combined to model an outcome, this is sometimes referred to as a “black box” model [48]. When it comes to healthcare applications and intervention design, having an “explainable” model where the mechanisms of recommendations can be understood may promote more trustworthiness among clinicians, end users and communities [49,50]. This approach of explainable AI models aligns with the principles of patient-centered care and recognizes that effective disaster preparedness strategies must be tailored to individuals’ specific circumstances to yield the highest impact [43]. Comprehensive datasets, like the Federal Emergency Management Agency (FEMA) *National Household Survey*, offer a valuable opportunity to apply ML approaches to identify the relative importance of factors associated with household disaster preparedness behaviors.

Tailoring preparedness interventions to at-risk communities bearing a disproportionate burden of adverse impacts from disasters, particularly those in historically redlined districts and impacted by structural racism, offers multiple advantages. By acknowledging the historical injustices and systemic disparities that have disproportionately impacted these communities, tailored interventions can provide a more holistic and empathetic approach, ideally based on trust and engagement, while addressing the root causes of vulnerability [43]. Firstly, customization ensures that these interventions are culturally sensitive and relevant to the unique preferences, needs and challenges of these communities. Secondly, customization helps bridge the equity gap. Communities impacted by structural racism often face higher levels of poverty, inadequate access to quality healthcare, and limited educational opportunities and increasing disproportionate risk from disasters [51]. Finally, customized interventions that address the inadequate infrastructure, healthcare access, and economic opportunities can simultaneously mitigate other risks that stem from macro-level inequities, enhancing resilience and health equity beyond disaster preparedness contexts [52]. By focusing on the unique challenges and strengths of each community, tailored interventions empower communities, promote inclusivity and equity, ultimately creating a more resilient and thriving society for all [53]. 

Overall, there is little high-quality evidence on the efficacy of current household disaster preparedness interventions, and tailored interventions are warranted by the state of the science [4]. This study seeks to address the gaps in the disaster preparedness literature by examining disparities in disaster preparedness among older adults (greater than 65 years old) who identify as African American/Black, participants of all ages who identify as African American/Black, older adults of all races and ethnicities, and all participants combined. The exploration of these diverse groups was intended to shed light on both group-specific and common factors that play crucial roles in shaping household disaster preparedness levels.

### Objective

The purpose here was to ascertain differences in the importance features of machine learning models of household disaster preparedness for older adults who identify as African American/Black, participants of all ages who identify as African American/Black, older adults of all races and ethnicities, and all participants combined to inform culturally tailored intervention recommendations for nursing practice. 

## 2. Materials and Methods

### 2.1. Design

We developed and tested a machine learning model from the publicly available 2018, 2019, and 2020 FEMA *National Household Survey* (NHS) datasets combined. We used publicly available and de-identified data, and this study did not meet the definition of human subjects research outlined by the Revised Common Rule (45 CFR §46) in the U.S. Therefore, no human subjects ethical review or approval was required. The original survey design is described elsewhere [54]. Briefly, the telephone survey is administered annually in a cross-sectional design from a nationally representative sampling frame combined with purposeful, non-probability oversampling from households in regions with higher risks for six customized hazards [54]. Our findings are reported according to the Strengthening the Reporting of Observational Studies in Epidemiology (STROBE) guidelines [55]. 

### 2.2. Setting

Survey participants were recruited from all 50 of the United States, the District of Columbia, Guam, Puerto Rico, and the U.S. Virgin Islands. Approximately half of the included participants were from the oversampling design in high-risk geographies [54]. Data were downloaded for this analysis from the OpenFEMA website on 26 September 2023. Survey data were collected by landline and mobile phone participant samples.

### 2.3. Participants

A total of the 15,048 participants’ data were included in the 2018 (*n* = 5003), 2019 (*n* = 5025), and 2020 (*n* = 5020) NHS datasets. Respondents must have a phone (landline or cell phone), be 18 years of age or older, and speak English or Spanish. Participants were randomly selected to participate in the NHS survey from population telephone sampling lists. Once the landline or mobile phone was answered, the interviewer requested to speak to the person who would have celebrated the next birthday in the household who was also available. We did not exclude any participants from the publicly available 2018–2020 datasets for analysis [54].

Our rationale for the years selected (2018–2020) was for the most congruence in survey questions, response options, customized hazards, and coding. The customized hazards for these years included: tornado, flood, hurricane, wildfire, earthquake, and urban event (nuclear explosion specifically for years 2018 and 2019, and 2020 is inclusive of, but not limited to, nuclear explosion). In contrast, the customized hazards for the 2021 NHS data included: drought, power outage, thunderstorm, tsunami, volcanic eruption, and winter storm; along with a heavy focus on pandemic-related survey items. In the 2017 NHS data, extreme heat and winter storm were also included, but urban event was not [54]. Thus, even though the 2017 and 2021 data were available, we excluded these datasets from the present analysis.

### 2.4. Variables

A total of 801 variables were available in the 2018 (*n* = 269), 2019 (*n* = 184), and 2020 (*n* = 348) NHS datasets [54]. Broadly, the survey included sections with items addressing disaster preparedness awareness, behaviors, financial preparedness, supplies, documents, information seeking, drills for all respondents, risk identification, efficacy, disaster experience, stages of preparedness, general disaster experience, and area-specific disaster questions/sections.

#### 2.4.1. Demographics

Demographic variables of sex/gender, age category, self-reported race (up to five responses allowed for bi/multiracial participants), ethnicity, primary language, education, home ownership, monthly household income, FEMA region, disability, and primary caregiving status, number of adults living in the household, and telephone usage were also included. For the purposes of our analyses, the responses for the five items assessing reported race were grouped into binary categories of (1) African American or Black, no other race reported, (2) White, no other race reported, (3) Asian, no other race reported, (4) American Indian or Alaska Native, no other race reported, and (5) Native Hawaiian or Pacific Islander, no other race reported. Biracial, multiracial, other, and those who did not report their race were designated as the referent group. 

#### 2.4.2. Readiness Score

The primary outcome variable for this work was a readiness score, with a possible range of 0–1. The score was composed of 12 survey items assessing the following:

Attended a meeting or training on preparedness.
Practiced emergency drill at home.
Made an emergency plan for household members.
Emergency plan includes evacuation routes.
Emergency plan includes checking on neighbors.
Plan for real-time alerts and warnings.
Supplies assembled for 3 days.
Supplies assembled for evacuation.
Critical documents safeguarded.
Communications plan with family members.
Insurance for residence.
Financial savings for an emergency.

Each item was scored as a 1 for “Yes” and 0 for “No”, except for the item addressing attending meetings or training. This item was scored with a 0.5 if the survey participant attended the training over a year ago and with a 1 if they attended the training within the last year. The mean score of non-missing items was used as the final readiness score. 

#### 2.4.3. Remaining Explanatory Variables

All of the remaining variables were eligible for data cleaning and harmonization across the three years for machine learning model development. 

### 2.5. Data Sources/Measurements

As described above, the NHS is administered annually by FEMA to monitor disaster preparedness progress among the American public [54]. While responses included weights by key demographic variables of age, gender, race/ethnicity, and education in order to enhance representativeness, our study utilized unweighted raw data. Our rationale was that our objectives aligned with ascertaining differences in feature priority for machine learning models by race, and our objective did not focus on population representativeness in the present analysis. 

### 2.6. Bias

Aligned with the objectives of this analysis, the nature of our aggregated and disaggregated analyses was designed to address the potential for data racial and age group bias. 

### 2.7. Study Size

All individual participant responses were considered for this analysis from the 2018–2020 annual NHS datasets [54].

### 2.8. Statistical Methods

The main analysis utilized in this study was the training and testing of random forest models for the aggregated and disaggregated groupings described in more detail below. A randomly generated 70/30 split created the training and test dataset. Analyses were conducted in STATA (17) and R Statistical Software (v4.3.0 “Already Tomorrow”) [56,57].

The data were prepared as follows. The 2018–2020 annual NHS datasets were appended to create a single dataset. Each variable was reviewed for harmonization across the three years of data, creating qualitative labels for all response sets. Continuous explanatory variables of age and income were grouped into categories. Nuclear and urban event data were harmonized as a single variable. We utilized the one-hot function, which generated binary variables for all qualitative response options. We utilized the sparsify function, which replaced missing data with zero values, altering the interpretation of 1 = yes and 0 = no to 1 = endorsed and 0 = not endorsed. The median was imputed for missing values in response to the item “How many days do you think you could last in your home without power, running water, or transportation?” If all 12 of the items that comprised the outcome score were missing, the participant’s data were excluded from the final model analysis. Outcome score differences between disaggregated groups were quantified using the Mann–Whitney U test. 

We looked at four groups of sampled participants based on age and self-reported racial identity: participants of all ages who identify as African American or Black, participants who identify as older adults and African American or Black, older adults of all races and ethnicities, and all participants combined. Between these groups, we looked at the top 40 variables of most importance in feature ranking for the model and outcome using the RMSE. Feature importance ranking is a machine learning task that measures the contributions of different variables to the performance of a specific model and outcome [58]. In this paper, we were looking to ascertain which variables were most important and/or strongly associated with household preparedness, and aimed to ascertain how these variables may differ among the four aforementioned groups. 

## 3. Results

### 3.1. Participants

After data cleaning and harmonization, a total of 252 variables from 15,048 participants were included. After all of the categorical responses were recorded to separate binary variables, 991 variables were included for analysis. Seven observations were dropped for having all data missing from the items used to compose the outcome score. One variable was excluded for no variability. Table 1 lists the descriptive statistics for the demographic variables of the sample. Additional demographic variables of home ownership, FEMA region, number of adults living in the household and telephone usage (cell or landline) are reported in an online Supplement Table S1. 

### 3.2. Outcome Preparedness Score

A mean of 0.58 (SD = 0.22) was observed for the preparedness score outcome for the whole sample (*n* = 15,041). Disaggregated by the racial grouping, the preparedness score outcome mean was 0.55 (SD = 0.24) for those who endorsed African American or Black racial identities (*n* = 1534) and mean of 0.58 (SD = 0.22) for those who did not endorse this identity (*n* = 13,507). We calculated a 53% chance that those who did not endorse having an African American/Black racial identity would have a higher preparedness score than those who did endorse an African American or Black identity (z = 4.599, *p* < 0.0001). Participants aged 65 or older with African American/Black identity demonstrated similar preparedness scores to other participants with Black/African American identity (m= 0.55, SD = 0.24, *n* = 368). Participants younger than 65 years old had a lower mean preparedness score (m = 0.57, SD = 0.23, *n* = 10,487) compared to those 65 or older (m = 0.60, SD = 0.21, *n* = 4554) with a 47% chance of being less prepared (z = −6.375, *p* < 0.0001). 

### 3.3. Main Results

Table 2 depicts the top 40 importance features by those with African American/Black identities, older adults with African American/Black identities and all older adults (Online Supplement Table S2 contains a color version of this table; Online Supplement Tables S3–S6 contains importance features for all four groups separately). The model for all participants explained 66.24% of the variance in the outcome with 500 trees, 329 variables at each split (MSE for test data = 0.017). The model with data disaggregated for only those with African American/Black racial identities explained 64.09% of the variance in the outcome (MSE for test data = 0.021). The model for participants aged 65+ explained 65.52% of the variance (MSE for test data = 0.017). Lastly, the model with disaggregated data for those aged 65+ with African American/Black racial identities explained 58.61% of the variance (MSE for test data = 0.02). 

## 4. Discussion

The findings of this study illuminate disparities in disaster household preparedness among African American/Black participants compared to individuals of other reported racial/ethnicity identities. These results align with previous research highlighting racial disparities in disaster preparedness, as well as economic and social inequalities that contribute to differential preparedness [9] and demonstrate similarities in overall population financial, age, race/ethnic identity, and transportation access features to COVID-19 mortality models [37]. Our findings make an important contribution to the literature. We reveal that nationally, African American/Black individuals may demonstrate slightly lower overall household disaster preparedness that is likely not clinically relevant, even if it is statistically significant. We further demonstrate that the associated factors for household preparedness vary by age and racial identity, which can inform future tailored interventions. The higher proportion of African American/Black participants who experienced negative impacts of disaster echoes how minoritized populations disproportionately experience disaster-related impacts [59]. This suggests the importance of viewing disaster preparedness through an intersectionality lens, which widens the racial justice discourse for those impacted by anti-Black racism and ageism. Additionally, it adds a critical objective to the field of environmental sociology to further examine disaster preparedness with racialized and other minoritized identities. This warrants further research to increase equitable disaster planning, preparedness, response, and recovery. 

Overall, we found little difference among groups for the priorities of assembling disaster supplies and creating emergency communication and evacuation plans in general, which supports continued whole population and all-hazard approaches to motivate community members to make a household disaster plan. Our findings point to a potential difference in the importance of social interventions and talking to others about a preparedness plan for older adults that supports further research for age-group tailoring in household disaster preparedness planning. This finding is novel compared to the background literature reviewed as a variable rarely considered, and advances previous research in the field [4,37]. The environmental justice movement addresses inequalities in disaster preparedness [60]. Environmental justice is an underlying shared value that motivates activities to eliminate societal inequalities that lead to a disproportionate burden of environmental exposures among at-risk populations. Environmental justice further informs the examination of historical inequalities that lead to differences in geographic mobility and resource access associated with cumulative and persistent adverse impacts of disasters [60]. Therefore, at its core, environmental justice values are aligned with the aims and goals as disaster preparedness movements. By including the social dimension of age in disaster research, age-specific results can help bolster requests to municipalities or governmental agencies to provide shelters and other disaster plans that are tailored to older adults and their specific needs. Our main findings discussed in detail below address financial and insurance preparedness disparities, information seeking, transportation, considerations for disabilities and life experience among older adults, and hazard specific tailoring opportunities. 

### 4.1. Financial and Insurance Preparedness

Our outcomes underscore the multifaceted nature of barriers to disaster preparedness faced by African American/Black participants. The identification of cost as a significant barrier is consistent with the previous historical literature demonstrating economic injustice and constraints that hinder disaster planning and resource allocation [61]. The lower reported median monthly household income among African American/Black participants, a finding corroborated by several recent studies [62,63,64,65,66], suggests financial limitations impact the ability to allocate preparedness resources. The substantially lower median disaster savings among African American/Black participants emphasizes the need for targeted interventions addressing economic justice, financial resilience, and equitable resource access.

Financial preparedness is crucial to addressing disaster-related health disparities. The findings from this study indicate that “no savings” was a priority feature for older adults with African American/Black identities, as was the preference not to answer how much they had in savings and their flood insurance status. Research indicates that in African American/Black households, lack of trust (which is deeply rooted in historically racist policies and structural barriers), is a cultural factor that influences financial preparedness decisions [65]. This may explain why many participants in this study chose not to answer how much they had in savings. Future research should focus on exploring how trust, stigma, and privacy concerns affect financial preparedness barriers for African American/Black communities.

African American/Black households report less knowledge of retirement planning and social security benefits, and also report feeling less financially prepared for retirement compared to white households [67]. Considering that families where the people with adult financial responsibilities to the household identify as African American/Black are more likely to have consumer debt and less likely to have housing debt [63], financial preparedness interventions should focus on protection of material assets by obtaining homeowners or rental insurance and flood insurance. Interventions should also address the importance of reducing or eliminating debt prior to retirement so that disaster-related expenses are more easily accommodated. As African American/Black identifying households are less willing to take financial risks and more likely to utilize a financial planner than White-identifying households [66], financial preparedness for disasters as a way to reduce asset risk should be included in discussions with financial planners.

Those with African American/Black racial identities have been/are disproportionately affected by mass incarceration, especially from the 1980s to the early 2000s. Research indicates that young adult and middle-aged formerly incarcerated African American/Black men are less able to set savings aside for any purpose and many do not have access to employer-provided pensions [62]. Nurses should plan and implement referrals to assist low-income African American/Black seniors with registering for the Supplemental Security Income Program, which, in addition to social security benefits, aids elderly community members with little income and few assets with a monthly stipend of $740 in 2018 [62].

The racial wealth gap is rooted in socioeconomic and political structure barriers [67], and financial preparedness is a priority to address health disparities. Community health and advanced practice nurses should consider offering group financial literacy/retirement planning classes to their clients at their practice sites, including disaster preparedness as a component. Research has shown that African American/Black community members are more likely to seek financial preparedness information from social services, including venues where nurses are present, such as senior centers and medical care offices [65]. Nurses can explore the differential impact of financial preparedness outreach interventions across race, ethnicity, gender, immigrant, and socioeconomic status, as well as prioritize the design, implementation, and evaluation of financial readiness interventions. 

### 4.2. Information about Disasters

One of our most important findings to inform public health and clinical practice involves information seeking and modalities of receiving disaster-related information and alerts. While information seeking in general was similar in all models, older adults with Black racial identities were the only group where information from TV, TV news, and the Weather Channel were entered as a priority feature associated with household disaster preparedness. Similarly, Black respondents in another study reported higher odds of confidence in their ability to attain health information and trust of health information from radio and television than other racial identities [68]. This digital divide in the utilization of the internet for information extends beyond seeking information related to disasters to health information seeking of any kind, with Black Americans, males, and those with lower socioeconomic status being the biggest predictors of never using the internet [69]. This digital divide exists among many demographic groups, including those with and without disabilities where people without disabilities were more likely to be aware of and use digital services during the COVID-19 than those with disabilities [67]. Rurality, income, age, and education level all have negative correlations with internet access (Federal Communications Commission, 2021) [70], exacerbating the digital divide. To ensure equal access and inclusiveness in the current era where so much is delivered electronically to improve health, the electronic literacy of disadvantaged groups should be addressed to help avoid increasing the digital divide [71]. Further, all participants in the current study with African American/Black racial identities and older adults with African American/Black racial identities reported they received no information was an importance feature only in their respective models, and any information received did not inform their steps to prepare for a disaster. It may be that the information they received did not inform their steps to prepare for a disaster due to social structural factors arising from historical, political, and cultural inequities and governmental policies that exacerbate inequities [72]. 

The modalities by which African American/Black communities receive disaster preparedness information requires further attention in practice, policy, and research. Approaches to improve disaster preparation might necessarily include enhancing trust and overcoming social structural factors and cultural inequities by delivering information designed with cultural humility, cultural relevance, and language preferences through trusted outlets [68]. Community-engaged disaster interventions culturally tailored to communities may be instrumental in addressing social vulnerability and enhancing the capacity for those with African American/Black racial identities at the local level [73].

### 4.3. Transportation

Transportation is a key social determinant of health and survival for disaster-affected individuals. Public transportation systems are vulnerable to the forces of disasters, particularly natural disasters. Transportation vulnerability has been defined in the literature multiple ways, including its susceptibility to interruptions or degradation that would decrease efficiency, service to users, and fragility of transport networks [74,75,76]. We found that, for those with Black/African American racial identities and older adults, a priority feature associated with household disaster preparedness was reliance on public transportation or the local authorities in order to leave a disaster-affected area than other groups. Alterations in public transportation can increase mortality and/or morbidity of disaster-affected individuals. Public transportation systems being damaged in disasters leads to decreased accessibility for rescue efforts and decreased connectivity to emergency aid/supplies [77]. Many geographical locations become less accessible after extreme weather events due to public transportation failure [78,79]. Current research on disaster transportation does not address the complex social needs of those who are most affected by disaster-related transport failure [77,80,81]. Future research should focus on the sociological aspects of transportation to inform policy and best support those at risk for transportation failure during the entire disaster cycle. 

### 4.4. Older Adults

Older adults, particularly those with multiple chronic health conditions or functional needs, are at high risk for deleterious health effects following a disaster [82,83]. Specific needs to post-disaster healthcare vary by disease, although challenges accessing care are common and contribute to poor health outcomes [84]. The focus of healthcare post-disaster is typically on immediate needs rather than on chronic conditions, heightening the risk for exacerbations that contribute to hospitalization and death. Disrupted/limited healthcare access, disrupted living patterns, and challenges related to obtaining medications and treatments contribute to increased hospitalizations [82]. Nurses have an opportunity to limit the negative health effects from disasters through astute attention toward chronic conditions and targeting interventions to mitigate factors that exacerbate them. Maintaining health and social resources are key elements of resilience, and interventions should consider the individual’s social roles and interactions as well as perceptions of their personal health status [85,86]. To maximize the effectiveness of disaster planning, nurses should support older adults in developing high-quality, individualized plans and encourage them to discuss content details with their family or social network [85,87]. 

Another beneficial approach to enhance the health and resilience of older adults post-disaster is to shift the focus from deficits to a strengths-based approach. Older adults have knowledge and resilience gained from experience—including disaster experience. Our study found experience with disasters was a priority importance feature for those 65+ and those 65+ with African American/Black racial identities. This finding was expected, as those who are older have more years to gain experience. Ultimately, older adults can share their experience to benefit members of their social network or the larger community [85,86,88,89]. Older adults often express greater interest in contributing to the well-being of others within their network than they have concern for themselves, thus engaging in personal preparedness activities can be presented as a means to aid others [85]. Methods to build personal and community resilience can often be most effective when they are “everyday activities” such as volunteering, participating in a group, such as book or craft club, or being involved with collaborative decision making [89]. Nurses should be alert for opportunities to capitalize on the experience of older adults living in the community. 

### 4.5. Hazard-Specific Interventions

In this study, major snowstorms and tornados were priority importance features to the household preparedness score in older adults with African American/Black racial identities. Families with more financial resources have the option of moving to areas more protected from hazards, while “socially vulnerable” or minoritized groups may have fewer choices [90]. However, disasters like tornadoes and snowstorms are unpredictable and impact locations indiscriminately in high-risk regions of the United States [91]. Older adults, particularly those that live alone and require medical equipment and electricity, are at increased risk for harm from disasters [15]. Our study results provide an opportunity to improve emergency preparedness nationally in this population, targeting hazards that are of particular importance to them, yet resulting in an improved overall emergency preparedness for all disaster types.

Older age, having experienced damage from a tornado, and having experienced an active tornado are three of the most decisive factors in individual preparedness for tornadoes [92]. Trust in the local and federal government were also important decisive factors in individual preparedness for tornadoes, with the former more important than the latter. As with disseminating information about disasters, information regarding emergency preparedness actions might best be achieved by improving trust in the information sought and overcoming social structural factors and cultural inequities by being designed with cultural humility, cultural relevance, and language preferences, and delivering them through trusted outlets [68]. Designated consistently as a highly trusted profession by the public, nurses are essential to providing disaster preparedness information in the programs and organizations they lead, in their individual health teaching, and patient care discharge instructions. 

After a snowstorm, mobility is of critical importance to access resources like food, medicine, or medical assistance. Research demonstrates that during and after the day of Snowstorm Uri in Texas, there was a decline in movement before gradually recovering [93]. However, fluctuations in recovery suggest that some areas were more impacted than others, influencing access to resources, emergency preparedness, and recovery for households of low socioeconomic status [93]. Further, in this study, elderly people appeared to be less resilient to disasters [93]. 

Though emergency preparedness for tornadoes and snowstorms is important across all demographics, emergency planners and responders must craft synchronized, well-ordered assistance which might vary across socioeconomic levels to support a social justice imperative and address the needs of more vulnerable populations in the mitigation and planning phases of a disaster [94].

### 4.6. Implications for Policy, Future Research and Clinical/Public Health Practice

The identified disparities in disaster preparedness among African American/Black participants hold implications for policy, practice, and further research. Policymakers should recognize the need for interventions aimed at reducing financial barriers to preparedness among marginalized communities. Community-based disaster preparedness programs should be tailored to address economic disparities, foster financial literacy, and provide accessible resources to facilitate saving for disaster readiness. Nursing and healthcare practitioners must be attuned to the unique challenges faced by African American/Black communities and provide culturally sensitive disaster education, encouraging proactive planning.

Further research is warranted to delve into the complex relationship between disaster preparedness and health among at-risk populations with a particular focus on household finances and transportation as key social determinants of health for disaster preparedness. Future investigations could explore the potential impact of disparities in disaster preparedness on health outcomes. The analysis reported here utilized a national dataset, and additional data collection to tailor the research with specific, smaller communities may be warranted. Additionally, qualitative research could provide deeper insights into the cultural, social, and structural factors influencing disaster preparedness decisions among African American/Black participants to inform meaningful cultural tailoring of clinical practice interventions, public health practice, and policies.

The artificial intelligence and machine learning methods utilized here may be replicated and refined in future research to continue to inform implications for practice. The continued need for explainability of AI/ML in nursing research is an important equity consideration so that nurses practicing in settings with limited AI/ML access and tools can apply the human understanding of the advances informed by AI/ML tools in better resourced and technology-assisted practice settings. We recommend the priority features of all AI/ML predictive models be well-explained and transparent to clinicians in order to enhance teamwork with AI-powered tools to improve both entirely human and AI-assisted clinical and public health performance. 

Lastly, future research is needed to more closely link household disaster preparedness with success in disaster response and health equity, mortality, morbidity, cost savings, and quality of life outcomes after disasters. The purpose of the research completed here was to ascertain differences in the importance features of machine learning models of household disaster preparedness for older adults who identify as African American/Black, participants of all ages who identify as African American/Black, older adults of all races and ethnicities, and all participants combined to inform culturally tailored intervention recommendations for nursing practice. The findings from this hypothesis-generating research reported here are being used to develop and refine a core hypothesis and testable research question for the next phase of this research. 

### 4.7. Generalizability and Limitations

Utilizing a national sample of surveys, the results of our study are generalizable to the survey-responding population of the United States and territories. Due to our unweighted analysis, the sample and findings are not representative of the population as a whole. In addition, our machine learning methods should be considered hypothesis generating and future work is needed to test and confirm the priority features we identified are causally linked to subsequent household preparedness levels.

Several limitations merit consideration. The cross-sectional nature of the data prevents establishing causal relationships between demographic factors, other priority features, and disaster preparedness. The use of self-reported income and savings data might introduce reporting bias and may not fully capture the financial complexities of participants. Additionally, the study focused solely on racial/ethnic disparities of African American/Black people and older adults, not accounting for other potential dimensions of discrimination, such as gender, disability, or health status. Aligned with our research questions, we treated items from branching logic on the survey as independent items, and further methodological adjustments in future research may be warranted to group or transform branching logic survey items during data pre-processing. 

### 4.8. Conclusions

In conclusion, this study advances our understanding of the disparities in disaster household preparedness among African American/Black participants, emphasizing the significance of economic constraints as a barrier. This is consistent with findings from previous emergency preparedness research demonstrating economic constraints are a significant barrier to emergency preparedness [61,94], necessitating a social justice perspective to address such inequities [94], particularly in light of research supporting evidence of a higher proportion of minoritized populations disproportionately experiencing disaster-related impacts [59]. Addressing these disparities requires multifaceted interventions involving economic justice, financial resilience, and equitable resource access, at the policy, community, and healthcare levels, aimed at promoting equity and resilience in the face of disasters. 

Our findings support the need for upstream interventions like financial preparedness delivered in a culturally appropriate manner by knowledgeable, trusted individuals in the African American/Black community to educate the community regarding the importance of protecting material assets, and ultimately reduce asset risk after a disaster. Nurses are uniquely positioned within communities and consistently trusted by the public [95] and should be leveraged to lead disaster preparedness and financial literacy education. Nurses can discharge these duties as a part of patient education anywhere, including churches, community centers, during home health visits, and in the acute care setting. 

Finally, emergency planners and policy makers would do well to acknowledge the digital divide that currently exists and better leverage a variety of modes of communication, including in person (by trusted individuals), television, and radio to educate African American/Black individuals in meaningful, engaging, and culturally relevant ways regarding household disaster preparedness, particularly in light of evidence that being a Black American, male, and of lower socioeconomic status significantly predicts never using the internet [69]. To be sure, the electronic literacy of disadvantaged groups should be addressed to help avoid increasing the digital divide [71]. 

As AI and ML continue to develop in terms of complexity and capability, researchers, emergency planners, and policy makers can gain critical insights to tailor disaster preparedness interventions to reduce health and financial disparities. ML has implications in identifying at-risk populations, customizing intervention strategies, aiding in the prioritization and equitable distribution of resources, addressing health disparities, and facilitating ongoing evaluation of disaster preparedness interventions by analyzing real-time data on their effectiveness, ensuring that interventions remain responsive to the evolving needs of diverse populations. By leveraging insights from ML-generated priority features in the FEMA National Household Survey data, nurses and other healthcare professionals can develop targeted and culturally sensitive interventions that account for the unique needs and concerns of various demographic groups. 

## Figures and Tables

**Table 1:** Demographic variables for the 2018, 2019, and 2020 National Household Surveys.

Demographic Characteristics	*n*	%
Sex or gender		
Female	7090	47.12
Male	7759	51.56
Other/Prefer to self-identify	33	0.22
Don’t know	18	0.12
Refused	148	0.98
Age category, years		
<30	2110	14.02
30–44	3038	20.19
45–64	5342	35.50
65–74	2495	16.58
>75	2063	13.71
Self-reported racial identity		
Asian	455	3.02
American Indian/Alaska Native	245	1.63
Black	1534	10.19
Native Hawaiian/Pacific Islander	143	0.95
White	10,042	66.73
Ethnicity: Hispanic, Latino or of Spanish origin		
Yes	2992	19.88
No	11,628	77.27
Don’t know	50	0.33
Refused	378	2.51
Primary language: English		
Yes	13,071	86.86
No	1581	10.51
Don’t know	42	0.28
Refused	354	2.35
Education		
Less than high school diploma	883	7.19
High school degree or diploma	2743	22.34
Some college	3365	27.40
Technical/vocational school	757	6.16
College graduate	4169	33.95
Post graduate work or degree	2767	18.39
Don’t know	76	0.62
Refused	288	2.35
Monthly household income, USD		
Under $60	195	1.30
$60 TO $499	194	1.29
$500 to $999	535	3.56
$1000 TO $1999	1044	6.94
$2000 TO $2999	1055	7.01
$3000 TO $3999	921	6.12
$4000 TO $4999	934	6.21
$5000 TO $7499	1565	10.40
$7500 TO $9999	802	5.33
$10,000 TO $14,999	940	6.25
$15,000 TO $19,999	323	2.15
Don’t know	1288	8.56
Refused	4107	27.29
Disability status		
Yes	2699	17.93
No	11,992	79.69
Don’t know	72	<1%
Refused	285	0.02
Primary caregiving status		
Yes	2601	17.28
No	12,110	80.48
Don’t know	49	0.33
Refused	288	1.91

Abbreviations: USD, United States dollars.

**Table 2:** Top 40 importance features disaggregated by primary groups of interest using appended data from the 2018, 2019, and 2020 National Household Surveys [54].

Variable Description	Answer	Category	Importance Ranking by Group			
			Age 65+ and Black Racial Identity	Black Racial Identity	Age 65+	Total Sample
“Does your plan include information about how to leave your community for an evacuation?”	No plan	EMERGENCY PLANS	1	1	1	1
“Does your plan include information about where to shelter or a safe place you can stay in the event of a disaster?”	Yes	EMERGENCY PLANS	2	2	2	2
“Which of the following best represents your (perceived level of) preparedness?”	Been prepared for more than 1 year	STAGES OF PREPAREDNESS	3	3	3	3
“How recently have you talked with others in your community about getting prepared for a disaster?”	I have not done this	INFORMATION SEEKING	4	9	5	8
“Can you give me a ballpark figure for the amount you have set aside?”	No savings	FINANCIAL PREPAREDNESS	5	10	7	7
“How recently have you talked with others in your community about getting prepared for a disaster?”	Within the past year	INFORMATION SEEKING	6	16	8	14
“How many days do you think you could last in your home without power, running water, or transportation?”	No supplies	SUPPLIES	7	6	6	4
Which of the following best represents your (perceived level of) preparedness?	I am not prepared, but I intend to get prepared in the next six months	STAGES OF PREPAREDNESS	8	7	16	10
How recently have you sought information about preparedness?	Within the past year	INFORMATION SEEKING	9	8	11	9
“How recently have you sought information about preparedness?”	I have not done this	INFORMATION SEEKING	10	4	4	6
“In the event of a disaster that required you to leave your area, would you need to rely on public transportation or the local authorities for transportation in order to leave?”	Yes	EMERGENCY PLANS	11	24	-	29
“Do you have a flood insurance policy from the National Flood Insurance Program or from a private insurance company?”	No	FINANCIAL PREPAREDNESS	12	18	34	26
“How confident are you that you can take the steps to prepare for a disaster in your area?”	Extremely confident	EFFICACY-SELF-CONFIDENCE	13	-	18	13
“Do you have a flood insurance policy from the National Flood Insurance Program or from a private insurance company?”	Yes	FINANCIAL PREPAREDNESS	14	17	32	22
After receiving the information about how to get better prepared, did you take any steps to prepare for a disaster?	Participant did not answer	CORE-INFORMATION	15	26	-	-
“In the past six months, have you read, seen, or heard any information about how to get better prepared for a disaster?”	No	CORE-INFORMATION	16	15	20	31
Is there a reason you think you would not be able to take the steps to prepare?	Participant did not answer	EFFICACY-SELF-CONFIDENCE	17	-	19	21
“Can you give me a ballpark figure for the amount you have set aside?”	Refused	FINANCIAL PREPAREDNESS	18	29	36	38
“Does your plan include information about where to shelter or a safe place you can stay in the event of a disaster?”	No plan	EMERGENCY PLANS	19	14	9	12
“In the past year, have you practiced what to do in a disaster by participating in a disaster preparedness exercise or drill? At another community location?”	No	DRILLS FOR ALL RESPONDENTS	20	22	17	16
How did you get the information that you read, saw, or heard about getting better prepared for a disaster?	Participant did not answer	CORE-INFORMATION	21	25	22	30
“How did you get the information that you read, saw, or heard about getting better prepared for a disaster?”	TV, TV news, weather channels	STAGES OF PREPAREDNESS	22	-	-	-
“In the event of a disaster that required you to leave your area, would you need to rely on public transportation or the local authorities for transportation in order to leave?”	No	EMERGENCY PLANS	23	38	30	24
“Have you or your family ever experienced the impacts of a disaster?”	Yes	DISASTER EXPERIENCE	24	-	24	-
“In the past six months, have you read, seen, or heard any information about how to get better prepared for a disaster?”	Yes	CORE-INFORMATION	25	19	37	33
Thinking about preparing yourself for a disaster, have you developed and discussed an action plan with your family, that includes information about how to leave your community or where to shelter, and have set aside supplies such as, food, water, and other essentials that allow you to be self-sufficient for at least three days?	I have been prepared for more than a year and I continue preparing	STAGES OF PREPAREDNESS	26	22	-	-
Thinking about preparing yourself for a disaster, have you developed and discussed an action plan with your family, that includes information about how to leave your community or where to shelter, and have set aside supplies such as, food, water, and other essentials that allow you to be self-sufficient for at least three days?	I have been prepared for the last year	STAGES OF PREPAREDNESS	27	5	10	5
“In the past year, have you practiced what to do in a disaster by participating in a disaster preparedness exercise or drill? At work?”	No	DRILLS FOR ALL RESPONDENTS	28	13	23	20
“All areas of the country are subject to different types of disasters. Will you please name the types of disasters that would have the biggest impact where you live?”	Tornado	RISK IDENTIFICATION	29	-	-	-
“How confident are you that you can take the steps to prepare for a disaster in your area?”	Not at all confident	EFFICACY-SELF-CONFIDENCE	30	39	-	-
What motivated you to take these steps to become better prepared? Please tell me the main reason.	Participant did not answer	STAGES OF PREPAREDNESS	31	-	-	-
“In the past year, have you practiced what to do in a disaster by participating in a disaster preparedness exercise or drill? At another community location?”	Yes	DRILLS FOR ALL RESPONDENTS	32	29	15	19
“Do you have a disability or a health condition that might affect your capacity to respond to an emergency situation? (INTERVIEWER: IF NECESSARY, READ:) A mobility, hearing, vision, cognitive, or intellectual disability or physical, mental, or health condition.”	Yes	DEMOGRAPHICS	33	30	-	-
Which of the following best represents your (perceived level of) preparedness?	I am not prepared, but I intend to start preparing in the next year	STAGES OF PREPAREDNESS	34	12	33	18
Which of the following best represents your (perceived level of) preparedness?	I am not prepared, and I do not intend to prepare in the next year	STAGES OF PREPAREDNESS	35	-	21	30
“When did you or your family experience a disaster?”	No experience	DISASTER EXPERIENCE	36	-	-	-
“How much would taking steps to prepare, such as creating a household emergency plan, developing an evacuation and shelter plan, signing up for alerts and warning systems, or stocking up on supplies help you get through a disaster in your area?”	Somewhat	STAGES OF PREPAREDNESS	37	-	-	-
Thinking about preparing yourself for a disaster, have you developed and discussed an action plan with your family, that includes information about how to leave your community or where to shelter, and have set aside supplies such as, food, water, and other essentials that allow you to be self-sufficient for at least three days?	I am not prepared, but I intend to get prepared in the next six months	STAGES OF PREPAREDNESS	38	-	-	-
“What was the information that you read, saw, or heard about how to get better prepared for a disaster?”	No information	STAGES OF PREPAREDNESS	39	23	28	-
“All areas of the country are subject to different types of disasters. Will you please name the types of disasters that would have the biggest impact where you live?”	A major snowstorm	RISK IDENTIFICATION	40	-	-	-

Footnote: Importance features are numbered by most important to less important, 1–40. A dash indicates the variable was not included in a particular group’s top 40 importance features. The background shading indicates the ranking of importance features from most to least important (dark to light).

## Data Availability

The analysis of this data is solely the responsibility of the authors and does not necessarily represent the official views of the National Institutes of Health, FEMA, and the Federal Government. FEMA and the Federal Government cannot vouch for the data or analyses derived from these data after the data have been retrieved from the Agency’s website, https://www.fema.gov/about/openfema/data-sets/national-household-survey (accessed on 26 September 2023).

## Supplementary Materials

The following supporting information can be downloaded at: https://www.mdpi.com/article/10.3390/ijerph21050521/s1, Table S1. Additional demographic variables for the 2018, 2019 and 2020 National Household Surveys; Table S2. Top 40 importance features disaggregated by primary groups of interest using appended data from the 2018, 2019 and 2020 National Household Surveys; Table S3. Top 40 importance features for 65 Years and Older and Black Racial Identity using appended data from the 2018, 2019 and 2020 National Household Surveys; Table S4. Top 40 importance features for Black Racial Identity using appended data from the 2018, 2019 and 2020 National Household Surveys; Table S5. Top 40 importance features for 65 years and older using appended data from the 2018, 2019 and 2020 National Household Surveys; Table S6. Top 40 importance features for total sample using appended data from the 2018, 2019 and 2020 National Household Surveys.
