# Supplementary material for: Tailoring Household Disaster Preparedness Interventions to Reduce Health Disparities: Nursing Implications from Machine Learning Importance Features from the 2018–2020 FEMA National Household Survey
Source: Int J Environ Res Public Health. 2024 Apr 23;21(5):521. doi: 10.3390/ijerph21050521 (PMC11121406; doi:10.3390/ijerph21050521)
Supplement: Supplementary file 1 [file ijerph-21-00521-s001.zip › ijerph-2910252-supplementary.pdf]

We have six supplemental tables that show (1) additional demographic variables of home ownership, FEMA region, number of adults living in the household and telephone usage (cell or landline); (2) a color version of Table S2 that is in the manuscript and Tables S3–S6 the importance feature rank (number 1–40), variable, variable description, X.IncMSE, and IncNodePurity for all four groups separately (Black racial identity and age 65+, Black racial identity, age 65+, total sample) to provide complete information with Table S2.

Supplementary Table S1. Additional demographic variables for the 2018, 2019 and 2020 National Household Surveys [54].

Demographic characteristics	*n*	%
Rent/own home		
Rent	4296	28.55
Own	9769	64.92
Don't know	82	0.54
Refused	610	4.05
Other	291	1.93
FEMA region		
1	320	2.13
2	1132	7.57
3	1032	6.86
4	3,390	22.53
5	1231	8.18
6	2078	13.81
7	513	3.41
8	722	4.80
9	3656	24.30
10	727	4.83
Missing	247	1.64
Number of adults living in the household		
1	3,668	24.38
2-5	10,695	71.07
6-10	193	1.28

11-20	25	0.17
21-30	5	0.03
31-40	2	0.01
>41	17	0.11
Telephone usage		
Cell phone		
Yes	14130	93.90
No	779	5.18
Don't know	9	0.06
Refused	130	0.86
Landline		
Yes	6594	43.82
No	8088	53.75
Don't know	40	0.27
Refused	326	2.17

Abbreviations: USD, United States Dollars; FEMA, Federal Emergency Management Agency.

Supplementary Table S2. Top 40 importance features disaggregated by primary groups of interest using appended data from the 2018, 2019 and 2020 National Household Surveys [54].

Variable Description	Answer	Category	Importance Ranking by Group			
			Age 65+ and Black Racial Identity	Black Racial Identity	Age 65+	Total Sample
"Does your plan include information about how to leave your community for an evacuation?"	No plan	EMERGENCY PLANS	**1**	1	1	1
"Does your plan include information about where to shelter or a safe place you can stay in the event of a disaster?"	Yes	EMERGENCY PLANS	**2**	2	2	2
"Which of the following best represents your (perceived level of) preparedness?"	Been prepared for more than 1 year	STAGES OF PREPAREDNESS	**3**	3	3	3
"How recently have you talked with others in your community about getting prepared for a disaster?"	I have not done this	INFORMATION SEEKING	**4**	9	5	8
"Can you give me a ballpark figure for the amount you have set aside?"	No savings	FINANCIAL PREPAREDNESS	**5**	10	7	7
"How recently have you talked with others in your community about getting prepared for a disaster?"	Within the past year	INFORMATION SEEKING	**6**	16	8	14

"How many days do you think you could last in your home without power, running water, or transportation?"	No supplies	SUPPLIES	**7**	6	6	4
Which of the following best represents your (perceived level of) preparedness?	I am not prepared, but I intend to get prepared in the next six months	STAGES OF PREPAREDNESS	**8**	7	16	10
How recently have you sought information about preparedness?	Within the past year	INFORMATION SEEKING	**9**	8	11	9
**"**How recently have you sought information about preparedness?"	I have not done this	INFORMATION SEEKING	**10**	4	4	6
"In the event of a disaster that required you to leave your area, would you need to rely on public transportation or the local authorities for transportation in order to leave?"	Yes	EMERGENCY PLANS	**11**	24	-	29
"Do you have a flood insurance policy from the National Flood Insurance Program or from a private insurance company?"	No	FINANCIAL PREPAREDNESS	**12**	18	34	26
"How confident are you that you can take the steps to prepare for a disaster in your area?"	Extremely confident	EFFICACY - SELF-CONFIDENCE	**13**	-	18	13

"Do you have a flood insurance policy from the National Flood Insurance Program or from a private insurance company?"	Yes	FINANCIAL PREPAREDNESS	**14**	17	32	22
After receiving the information about how to get better prepared, did you take any steps to prepare for a disaster?	Participant did not answer	CORE - INFORMATION	**15**	26	-	-
"In the past six months, have you read, seen, or heard any information about how to get better prepared for a disaster?"	No	CORE - INFORMATION	**16**	15	20	31
Is there a reason you think you would not be able to take the steps to prepare?	Participant did not answer	EFFICACY - SELF-CONFIDENCE	**17**	-	19	21
"Can you give me a ballpark figure for the amount you have set aside?"	Refused	FINANCIAL PREPAREDNESS	**18**	29	36	38
"Does your plan include information about where to shelter or a safe place you can stay in the event of a disaster?"	No plan	EMERGENCY PLANS	**19**	14	9	12
"In the past year, have you practiced what to do in a disaster by participating in a disaster preparedness exercise or drill? At another community location?"	No	DRILLS FOR ALL RESPONDENTS	**20**	22	17	16

How did you get the information that you read, saw, or heard about getting better prepared for a disaster?	Participant did not answer	CORE - INFORMATION	**21**	25	22	30
"How did you get the information that you read, saw, or heard about getting better prepared for a disaster?"	TV, TV news, weather channels	STAGES OF PREPAREDNESS	**22**	-	-	-
"In the event of a disaster that required you to leave your area, would you need to rely on public transportation or the local authorities for transportation in order to leave?"	No	EMERGENCY PLANS	**23**	38	30	24
"Have you or your family ever experienced the impacts of a disaster?"	Yes	DISASTER EXPERIENCE	**24**	-	24	-
"In the past six months, have you read, seen, or heard any information about how to get better prepared for a disaster?"	Yes	CORE - INFORMATION	**25**	19	37	33

Thinking about preparing yourself for a disaster, have you developed and discussed an action plan with your family, that includes information about how to leave your community or where to shelter, and have set aside supplies such as, food, water, and other essentials that allow you to be self-sufficient for at least three days?	I have been prepared for more than a year and I continue preparing	STAGES OF PREPAREDNESS	**26**	22	-	-
Thinking about preparing yourself for a disaster, have you developed and discussed an action plan with your family, that includes information about how to leave your community or where to shelter, and have set aside supplies such as, food, water, and other essentials that allow you to be self-sufficient for at least three days?	I have been prepared for the last year	STAGES OF PREPAREDNESS	**27**	5	10	5
"In the past year, have you practiced what to do in a disaster by participating in a disaster preparedness exercise or drill? At work?"	No	DRILLS FOR ALL RESPONDENTS	**28**	13	23	20

"All areas of the country are subject to different types of disasters. Will you please name the types of disasters that would have the biggest impact where you live?"	Tornado	RISK IDENTIFICATION	**29**	-	-	-
"How confident are you that you can take the steps to prepare for a disaster in your area?"	Not at all confident	EFFICACY - SELF-CONFIDENCE	**30**	39	-	-
What motivated you to take these steps to become better prepared? Please tell me the main reason.	Participant did not answer	STAGES OF PREPAREDNESS	**31**	-	-	-
"In the past year, have you practiced what to do in a disaster by participating in a disaster preparedness exercise or drill? At another community location?"	Yes	DRILLS FOR ALL RESPONDENTS	**32**	29	15	19
"Do you have a disability or a health condition that might affect your capacity to respond to an emergency situation? (INTERVIEWER: IF NECESSARY, READ:) A mobility, hearing, vision, cognitive, or intellectual disability or physical, mental, or health condition.)"	Yes	DEMOGRAPHICS	**33**	30	-	-

Which of the following best represents your (perceived level of) preparedness?	I am not prepared, but I intend to start preparing in the next year	STAGES OF PREPAREDNESS	**34**	12	33	18
Which of the following best represents your (perceived level of) preparedness?	I am not prepared, and I do not intend to prepare in the next year	STAGES OF PREPAREDNESS	**35**	-	21	30
"When did you or your family experience a disaster?"	No experience	DISASTER EXPERIENCE	**36**	-	-	-
**"**How much would taking steps to prepare, such as creating a household emergency plan, developing an evacuation and shelter plan, signing up for alerts and warning systems, or stocking up on supplies help you get through a disaster in your area?"	Somewhat	STAGES OF PREPAREDNESS	**37**	-	-	-

Thinking about preparing yourself for a disaster, have you developed and discussed an action plan with your family, that includes information about how to leave your community or where to shelter, and have set aside supplies such as, food, water, and other essentials that allow you to be self-sufficient for at least three days?	I am not prepared, but I intend to get prepared in the next six months	STAGES OF PREPAREDNESS	**38**	-	-	-
"What was the information that you read, saw, or heard about how to get better prepared for a disaster?"	No information	STAGES OF PREPAREDNESS	**39**	23	28	-
"All areas of the country are subject to different types of disasters. Will you please name the types of disasters that would have the biggest impact where you live?"	A major snowstorm	RISK IDENTIFICATION	**40**	-	-	-

Footnote: Importance features are numbered by most important to less important, 1 - 40. A dash indicates the variable was not included in a particular group’s top 40 importance features. The background shading indicates the ranking of importance features from most to least important (green to red).

Supplementary Table S3. Top 40 importance features for 65 Years and Older and Black Racial Identity using appended data from the 2018, 2019 and 2020 National Household Surveys [54].

Importance Feature Rank	Variable for 65 Years and Older and Black Racial Identity	Variable Description	X.IncMSE	IncNodePurity
**1**	b6_1_	Does your plan include information about how to leave your community for an evacuation?	0.014329438	2.432309016
**2**	b6_1_YES	Does your plan include information about where to shelter or a safe place you can stay in the event of a disaster?	0.012119855	1.961971329
**3**	st_stg1_I.HAVE.BEEN.PREPARED.FOR.MORE	Which of the following best represents your (perceived level of) preparedness?	0.002491092	0.564541965
**4**	b2b_I.HAVE.NOT.DONE.THIS	How recently have you talked with others in your community about getting prepared for a disaster?	0.001945243	0.366684511
**5**	fp4_1_	Can you give me a ballpark figure for the amount you have set aside?	0.001274035	0.230091212
**6**	b2b_WITHIN.THE.PAST.YEAR	How recently have you talked with others in your community about getting prepared for a disaster?	0.001218232	0.323881563
**7**	b10	How many days do you think you could last in your home without power, running water, or transportation?	0.001194423	0.359234314
**8**	st_stg1_I.AM.NOT.PREPARED..BUT.I.INTEND.TO.GE	Which of the following best represents your (perceived level of) preparedness?	0.000814904	0.171036438
**9**	b2c_WITHIN.THE.PAST.YEAR	How recently have you sought information about preparedness?	0.000749693	0.326522056

**10**	b2c_I.HAVE.NOT.DONE.THIS	How recently have you sought information about preparedness?	0.000736454	0.386149393
**11**	b6a_YES	In the event of a disaster that required you to leave your area, would you need to rely on public transportation or the local authorities for transportation in order to leave?	0.000454245	0.100502724
**12**	fp2_NO	Do you have a flood insurance policy from the National Flood Insurance Program or from a private insurance company?	0.000439624	0.136991809
**13**	c2_EXTREMELY.CONFIDENT	How confident are you that you can take the steps to prepare for a disaster in your area?	0.000414527	0.078636871
**14**	fp2_YES	Do you have a flood insurance policy from the National Flood Insurance Program or from a private insurance company?	0.000407221	0.093234709
**15**	a2b_	After receiving the information about how to get better prepared, did you take any steps to prepare for a disaster?	0.000399129	0.135750058
**16**	a1_NO	In the past six months, have you read, seen, or heard any information about how to get better prepared for a disaster?	0.000397055	0.106633887
**17**	c2a_	Is there a reason you think you would not be able to take the steps to prepare?	0.000352556	0.096210618
**18**	fp4_1_REFUSED	Can you give me a ballpark figure for the amount you have set aside?	0.000327386	0.090551415
**19**	b6_1_NO	Does your plan include information about where to shelter or a safe place you can stay in the event of a disaster?	0.000303314	0.118268234
**20**	b3d_NO	In the past year, have you practiced what to do in a disaster by participating in a disaster preparedness exercise or drill? At another community location?	0.00028385	0.06277185

**21**	a1a_orig201_	How did you get the information that you read, saw, or heard about getting better prepared for a disaster?	0.000276716	0.083573853
**22**	a1a_orig201_TV..TV.NEWS..WEATHER.CHANNELS	How did you get the information that you read, saw, or heard about getting better prepared for a disaster?	0.000264782	0.054280841
**23**	b6a_NO	In the event of a disaster that required you to leave your area, would you need to rely on public transportation or the local authorities for transportation in order to leave?	0.000254527	0.068826839
**24**	genexp1_YES	Have you or your family ever experienced the impacts of a disaster?	0.000224392	0.056588231
**25**	a1_YES	In the past six months, have you read, seen, or heard any information about how to get better prepared for a disaster?	0.000222214	0.096703329
**26**	hz_stg1_I.HAVE.BEEN.PREPARED.FOR.MORE	Thinking about preparing yourself for a disaster, have you developed and discussed an action plan with your family, that includes information about how to leave your community or where to shelter, and have set aside supplies such as, food, water, and other essentials that allow you to be self-sufficient for at least three days?	0.000210247	0.054308821
**27**	st_stg1_I.HAVE.BEEN.PREPARED.FOR.THE.LAST.YEAR	Thinking about preparing yourself for a disaster, have you developed and discussed an action plan with your family, that includes information about how to leave your community or where to shelter, and have set aside supplies such as, food, water, and other essentials that allow you to be self-sufficient for at least three days?	0.000193937	0.083559999
**28**	b3b_NO	In the past year, have you practiced what to do in a disaster by participating in a disaster preparedness exercise or drill? At work?	0.000190513	0.079868873
**29**	b101_A.TORNADO	All areas of the country are subject to different types of disasters. Will you please name the types of disasters that would have the biggest impact where you live?	0.000182762	0.108550993
**30**	c2_NOT.AT.ALL.CONFIDENT	How confident are you that you can take the steps to prepare for a disaster in your area? Would you say you are...	0.000177868	0.043192359

**31**	a1dcode_	What motivated you to take these steps to become better prepared? Please tell me the main reason.	0.000175014	0.058457287
**32**	b3d_YES	In the past year, have you practiced what to do in a disaster by participating in a disaster preparedness exercise or drill? At another community location?	0.000155029	0.069032167
**33**	d4a_YES	Do you have a disability or a health condition that might affect your capacity to respond to an emergency situation? (INTERVIEWER: IF NECESSARY, READ:) A mobility, hearing, vision, cognitive, or intellectual disability or physical, mental, or health condition.)	0.000144438	0.079222669
**34**	st_stg1_I.AM.NOT.PREPARED..BUT.I.INTEND.TO.ST	Which of the following best represents your (perceived level of) preparedness?	0.000143942	0.055080695
**35**	st_stg1_I.AM.NOT.PREPARED..AND.I.DO.NOT	Which of the following best represents your (perceived level of) preparedness?	0.000142557	0.06823186
**36**	genexp12_cat_	When did you or your family experience a disaster?	0.000134949	0.044472379
**37**	c1_SOMEWHAT	How much would taking steps to prepare, such as creating a household emergency plan, developing an evacuation and shelter plan, signing up for alerts and warning systems, or stocking up on supplies help you get through a disaster in your area? Would it help	0.00012865	0.076670593
**38**	hz_stg1_I.AM.NOT.PREPARED..BUT.I.INTEND.TO.GE	Thinking about preparing yourself for a disaster, have you developed and discussed an action plan with your family, that includes information about how to leave your community or where to shelter, and have set aside supplies such as, food, water, and other essentials that allow you to be self-sufficient for at least three days?	0.00010996	0.031844319
**39**	a1a_orig101_	What was the information that you read, saw, or heard about how to get better prepared for a disaster?	0.00010408	0.064222635
**40**	b101_A.MAJOR.SNOWSTORM	All areas of the country are subject to different types of disasters. Will you please name the types of disasters that would have the biggest impact where you live?	0.000102021	0.033420088

Supplementary Table S4. Top 40 importance features for Black Racial Identity using appended data from the 2018, 2019 and 2020 National Household Surveys [54].

Importance Feature Rank	Variable for Black Racial Identity	Variable Description	X.IncMSE	IncNodePurity
1	b6_1_	Does your plan include information about how to leave your community for an evacuation?	0.015537012	12.38820039
2	b6_1_YES	Does your plan include information about where to shelter or a safe place you can stay in the event of a disaster?	0.010056289	9.013570339
3	st_stg1_I.HAVE.BEEN.PREPARED.FOR.MORE	Which of the following best represents your (perceived level of) preparedness?	0.005095154	3.420727261
4	b2c_I.HAVE.NOT.DONE.THIS	How recently have you sought information about preparedness?	0.00159505	2.269248656
5	st_stg1_I.HAVE.BEEN.PREPARED.FOR.THE.LAST.YEAR	Thinking about preparing yourself for a disaster, have you developed and discussed an action plan with your family, that includes information about how to leave your community or where to shelter, and have set aside supplies such as, food, water, and other essentials that allow you to be self-sufficient for at least three days?	0.001282471	0.969962526
6	b10	How many days do you think you could last in your home without power, running water, or transportation?	0.001172069	1.30146007
7	st_stg1_I.AM.NOT.PREPARED..BUT.I.INTEND.TO.GE	Which of the following best represents your (perceived level of) preparedness?	0.000976308	0.66425605

**8**	b2c_WITHIN.THE.PAST.YEAR	How recently have you sought information about preparedness?	0.000846639	1.110468104
**9**	b2b_I.HAVE.NOT.DONE.THIS	How recently have you talked with others in your community about getting prepared for a disaster?	0.000824237	0.903501979
**10**	fp4_1_	How recently have you talked with others in your community about getting prepared for a disaster?	0.000673229	0.455127463
**11**	b3b_YES	Can you give me a ballpark figure for the amount you have set aside?	0.00060631	0.462409374
**12**	st_stg1_I.AM.NOT.PREPARED..BUT.I.INTEND.TO.ST	Which of the following best represents your (perceived level of) preparedness?	0.000550973	0.34540015
**13**	b3b_NO	In the past year, have you practiced what to do in a disaster by participating in a disaster preparedness exercise or drill? At work?	0.000502227	0.456670658
**14**	b6_1_NO	Does your plan include information about where to shelter or a safe place you can stay in the event of a disaster?	0.000433802	0.550972062
**15**	a1_NO	In the past six months, have you read, seen, or heard any information about how to get better prepared for a disaster?	0.000370463	0.369724993
**16**	b2b_WITHIN.THE.PAST.YEAR	How recently have you talked with others in your community about getting prepared for a disaster?	0.000332268	0.422733187
**17**	fp2_YES	Do you have a flood insurance policy from the National Flood Insurance Program or from a private insurance company?	0.000327096	0.400219008
**18**	fp2_NO	Do you have a flood insurance policy from the National Flood Insurance Program or from a private insurance company?	0.000266017	0.264669771

**19**	a1_YES	In the past six months, have you read, seen, or heard any information about how to get better prepared for a disaster?	0.000236728	0.554126054
**20**	d8_OWN	Do you rent or own your home?	0.000220788	0.34591855
**21**	b3d_NO	In the past year, have you practiced what to do in a disaster by participating in a disaster preparedness exercise or drill? At another community location?	0.000211931	0.217404088
**22**	hz_stg1_I.HAVE.BEEN.PREPARED.FOR.MORE	Thinking about preparing yourself for a disaster, have you developed and discussed an action plan with your family, that includes information about how to leave your community or where to shelter, and have set aside supplies such as, food, water, and other essentials that allow you to be self-sufficient for at least three days?	0.000204656	0.151260815
**23**	a1a_orig101_	What was the information that you read, saw, or heard about how to get better prepared for a disaster?	0.00019868	0.351854178
**24**	b6a_YES	In the event of a disaster that required you to leave your area, would you need to rely on public transportation or the local authorities for transportation in order to leave?	0.000174266	0.223773912
**25**	a1a_orig201_	How did you get the information that you read, saw, or heard about getting better prepared for a disaster?	0.000158448	0.386013755
**26**	a2b_	After receiving the information about how to get better prepared, did you take any steps to prepare for a disaster?	0.000155652	0.3672497
**27**	a2b_YES	After receiving the information about how to get better prepared, did you take any steps to prepare for a disaster?	0.00013695	0.293471783
**28**	fp4_1_REFUSED	Can you give me a ballpark figure for the amount you have set aside?	0.000120627	0.123772278

**29**	b3d_YES	In the past year, have you practiced what to do in a disaster by participating in a disaster preparedness exercise or drill? At another community location?	0.000119734	0.143672532
**30**	d4a_YES	Do you have a disability or a health condition that might affect your capacity to respond to an emergency situation? (INTERVIEWER: IF NECESSARY, READ:) A mobility, hearing, vision, cognitive, or intellectual disability or physical, mental, or health condition.)	0.000101654	0.154122375
**31**	d12_	Of all the telephone calls your household receives, would you say that...	9.70E-05	0.15034018
**32**	d4a_NO	Do you have a disability or a health condition that might affect your capacity to respond to an emergency situation?	9.68E-05	0.184665449
**33**	a1a_orig102_	What was the information that you read, saw, or heard about how to get better prepared for a disaster? (2nd response of up to 2)	9.31E-05	0.091245763
**34**	d2_POST.GRADUATE.WORK.OR.DEGREE	What is your highest completed level of education?	9.06E-05	0.142991207
**35**	b10_3_YES	Do you have supplies to stay for 24 hours at any other place you frequent?	8.51E-05	0.088726913
**36**	b3c_NO	In the past year, have you practiced what to do in a disaster by participating in a disaster preparedness exercise or drill? At school?	8.26E-05	0.137128628
**37**	d8_RENT	Do you rent or own your home?	7.64E-05	0.220243419
**38**	b6a_NO	In the event of a disaster that required you to leave your area, would you need to rely on public transportation or the local authorities for transportation in order to leave?	7.53E-05	0.208612461

**39**	c2_NOT.AT.ALL.CONFIDENT	How confident are you that you can take the steps to prepare for a disaster in your area? Would you say you are...	7.41E-05	0.102301228
**40**	genexp1_NO	Have you or your family ever experienced the impacts of a disaster?	7.40E-05	0.109840905

Supplementary Table S5. Top 40 importance features for 65 years and older using appended data from the 2018, 2019 and 2020 National Household Surveys [54].

Importance Feature Rank	Variable for 65 Years and Older	Variable Description	X.IncMSE	IncNodePurity
**1**	b6_1_	Does your plan include information about how to leave your community for an evacuation?	0.013248756	29.66588376
**2**	b6_1_YES	Does your plan include information about where to shelter or a safe place you can stay in the event of a disaster?	0.006230234	16.48670121
**3**	st_stg1_I.HAVE.BEEN.PREPARED.FOR.MORE	Which of the following best represents your (perceived level of) preparedness?	0.003214382	9.733042803
**4**	b2c_I.HAVE.NOT.DONE.THIS	How recently have you sought information about preparedness?	0.001220138	4.84434617
**5**	b2b_I.HAVE.NOT.DONE.THIS	How recently have you talked with others in your community about getting prepared for a disaster?	0.001078267	4.442502956
**6**	b10	How many days do you think you could last in your home without power, running water, or transportation?	0.001048985	3.420407207

**7**	fp4_1_	Can you give me a ballpark figure for the amount you have set aside?	0.000805563	1.645920884
**8**	b2b_WITHIN.THE.PAST.YEAR	How recently have you talked with others in your community about getting prepared for a disaster?	0.000716656	2.9591149
**9**	b6_1_NO	Does your plan include information about where to shelter or a safe place you can stay in the event of a disaster?	0.000544714	1.645149644
**10**	st_stg1_I.HAVE.BEEN.PREPARED.FOR.THE.LAST.YEAR	Thinking about preparing yourself for a disaster, have you developed and discussed an action plan with your family, that includes information about how to leave your community or where to shelter, and have set aside supplies such as, food, water, and other essentials that allow you to be self-sufficient for at least three days?	0.000532435	1.517311705
**11**	b2c_WITHIN.THE.PAST.YEAR	How recently have you sought information about preparedness?	0.000509239	2.060664747
**12**	d8_RENT	Do you rent or own your home?	0.000489978	1.676487067
**13**	d8_OWN	Do you rent or own your home?	0.000380223	1.496146224
**14**	a2b_YES	After receiving the information about how to get better prepared, did you take any steps to prepare for a disaster?	0.000341621	1.5922901
**15**	b3d_YES	In the past year, have you practiced what to do in a disaster by participating in a disaster preparedness exercise or drill? At another community location?	0.000288788	0.706061896
**16**	st_stg1_I.AM.NOT.PREPARED..BUT.I.INTEND.TO.GE	Which of the following best represents your (perceived level of) preparedness?	0.000288495	0.606678878

**17**	b3d_NO	In the past year, have you practiced what to do in a disaster by participating in a disaster preparedness exercise or drill? At another community location?	0.000260258	0.676830324
**18**	c2_EXTREMELY.CONFIDENT	How confident are you that you can take the steps to prepare for a disaster in your area?	0.000182186	0.779207871
**19**	c2a_	Is there a reason you think you would not be able to take the steps to prepare?	0.000167038	0.552462946
**20**	a1_NO	In the past six months, have you read, seen, or heard any information about how to get better prepared for a disaster?	0.000163696	0.562308401
**21**	st_stg1_I.AM.NOT.PREPARED..AND.I.DO.NOT	Which of the following best represents your (perceived level of) preparedness?	0.000120565	0.267402564
**22**	a1a_orig201_	How did you get the information that you read, saw, or heard about getting better prepared for a disaster?	0.000112047	0.437967556
**23**	b3b_NO	In the past year, have you practiced what to do in a disaster by participating in a disaster preparedness exercise or drill? At work?	9.86E-05	0.503816592
**24**	genexp1_YES	Have you or your family ever experienced the impacts of a disaster?	9.67E-05	0.254231734
**25**	d13_.1.000.TO..1.999	What is your total MONTHLY household income, before taxes? Please include income from wages and salaries, remittances from family members living elsewhere, farming, and all other sources.	9.45E-05	0.43095859
**26**	d3_YES	What is your total MONTHLY household income, before taxes? Please include income from wages and salaries, remittances from family members living elsewhere, farming, and all other sources.	9.45E-05	0.527183891

**27**	d15_YES	Is English the primary or main language spoken among those living in your household?	9.38E-05	0.338274784
**28**	a1a_orig101_	What was the information that you read, saw, or heard about how to get better prepared for a disaster?	9.26E-05	0.395361093
**29**	d11_NO	Do you have a cell phone that you use to make and receive personal calls?	9.08E-05	0.421546184
**30**	b6a_NO	In the event of a disaster that required you to leave your area, would you need to rely on public transportation or the local authorities for transportation in order to leave?	8.98E-05	0.55239174
**31**	d15_NO	Is English the primary or main language spoken among those living in your household?	8.82E-05	0.328019236
**32**	fp2_YES	Do you have a flood insurance policy from the National Flood Insurance Program or from a private insurance company?	8.73E-05	0.378851867
**33**	st_stg1_I.AM.NOT.PREPARED..BUT.I.INTEND.TO.ST	Which of the following best represents your (perceived level of) preparedness?	8.62E-05	0.356070063
**34**	fp2_NO	Do you have a flood insurance policy from the National Flood Insurance Program or from a private insurance company?	7.69E-05	0.376814452
**35**	d2_LESS.THAN.HIGH.SCHOOL.DIPLOMA	What is your highest completed level of education?	7.68E-05	0.470853045
**36**	fp4_1_REFUSED	Can you give me a ballpark figure for the amount you have set aside?	7.60E-05	0.375316859
**37**	a1_YES	In the past six months, have you read, seen, or heard any information about how to get better prepared for a disaster?	7.49E-05	0.489668742

**38**	b3b_YES	In the past year, have you practiced what to do in a disaster by participating in a disaster preparedness exercise or drill? At work?	7.47E-05	0.385918723
**39**	white_only	Describe your race.	7.24E-05	0.554590291
**40**	d13_.500.TO..999	What is your total MONTHLY household income, before taxes? Please include income from wages and salaries, remittances from family members living elsewhere, farming, and all other sources.	6.96E-05	0.408834697

Supplementary Table S6. Top 40 importance features for total sample using appended data from the 2018, 2019 and 2020 National Household Surveys [54].

Importance Feature Rank	Variable for Total Sample	Variable Description	X.IncMSE	IncNodePurity
**1**	b6_1_	Does your plan include information about how to leave your community for an evacuation?	0.013085328	111.2465689
**2**	b6_1_YES	Does your plan include information about where to shelter or a safe place you can stay in the event of a disaster?	0.005638424	64.88549302
**3**	st_stg1_I.HAVE.BEEN.PREPARED.FOR.MORE	Which of the following best represents your (perceived level of) preparedness?	0.005592052	46.27837258
**4**	b10	How many days do you think you could last in your home without power, running water, or transportation?	0.001700082	14.51498766

**5**	st_stg1_I.HAVE.BEEN.PREPARED.FOR.THE.LAST.YEAR	Which of the following best represents your (perceived level of) preparedness?	0.001621637	11.16030869
**6**	b2c_I.HAVE.NOT.DONE.THIS	How recently have you sought information about preparedness?	0.001111868	17.47013338
**7**	fp4_1_	Can you give me a ballpark figure for the amount you have set aside?	0.000898553	5.775548754
**8**	b2b_I.HAVE.NOT.DONE.THIS	How recently have you talked with others in your community about getting prepared for a disaster?	0.00088133	14.54051772
**9**	b2c_WITHIN.THE.PAST.YEAR	How recently have you sought information about preparedness?	0.000646657	7.421689142
**10**	st_stg1_I.AM.NOT.PREPARED..BUT.I.INTEND.TO.GE	Which of the following best represents your (perceived level of) preparedness?	0.000642929	4.276641999
**11**	d8_OWN	Do you rent or own your home?	0.000642021	7.497732906
**12**	b6_1_NO	Does your plan include information about where to shelter or a safe place you can stay in the event of a disaster?	0.000503619	5.114550837
**13**	c2_EXTREMELY.CONFIDENT	How confident are you that you can take the steps to prepare for a disaster in your area?	0.000396384	3.621858808
**14**	b2b_WITHIN.THE.PAST.YEAR	How recently have you talked with others in your community about getting prepared for a disaster?	0.000394878	6.301341763
**15**	a2b_YES	After receiving the information about how to get better prepared, did you take any steps to prepare for a disaster?	0.000322898	3.676677031

**16**	b3d_NO	In the past year, have you practiced what to do in a disaster by participating in a disaster preparedness exercise or drill? At another community location?	0.000315951	2.735221287
**17**	d8_RENT	Do you rent or own your home?	0.000288729	3.563784429
**18**	st_stg1_I.AM.NOT.PREPARED..BUT.I.INTEND.TO.ST	Which of the following best represents your (perceived level of) preparedness?	0.0002556	1.802560224
**19**	b3d_YES	In the past year, have you practiced what to do in a disaster by participating in a disaster preparedness exercise or drill? At another community location?	0.000231276	2.217080092
**20**	b3b_NO	In the past year, have you practiced what to do in a disaster by participating in a disaster preparedness exercise or drill? At work?	0.000205256	2.219402793
**21**	c2a_	Is there a reason you think you would not be able to take the steps to prepare?	0.000199315	2.3486113
**22**	fp2_YES	Do you have a flood insurance policy from the National Flood Insurance Program or from a private insurance company?	0.000193921	1.698002543
**23**	d3_YES		0.000174255	1.90190422
**24**	b6a_NO	In the event of a disaster that required you to leave your area, would you need to rely on public transportation or the local authorities for transportation in order to leave?	0.000145538	2.325690293
**25**	b3b_YES	In the past year, have you practiced what to do in a disaster by participating in a disaster preparedness exercise or drill? At work?	0.000145091	1.726271459
**26**	fp2_NO	Do you have a flood insurance policy from the National Flood Insurance Program or from a private insurance company?	0.000120436	1.475069881

**27**	d3_NO	Are you of Hispanic, Latino, or Spanish origin - such as Mexican, Puerto Rican, Cuban, or other Spanish origin?	0.000114981	1.483470179
**28**	d2_LESS.THAN.HIGH.SCHOOL.DIPLOMA	What is your highest completed level of education?	0.000112383	1.614758361
**29**	b6a_YES	In the event of a disaster that required you to leave your area, would you need to rely on public transportation or the local authorities for transportation in order to leave?	0.000110096	1.761719307
**30**	st_stg1_I.AM.NOT.PREPARED..AND.I.DO.NOT	Which of the following best represents your (perceived level of) preparedness?	0.000102038	0.855599747
**31**	a1_NO	In the past six months, have you read, seen, or heard any information about how to get better prepared for a disaster?	8.83E-05	1.046483147
**32**	d15_NO	Is English the primary or main language spoken among those living in your household?	7.68E-05	1.085714035
**33**	a1_YES	In the past six months, have you read, seen, or heard any information about how to get better prepared for a disaster?	7.42E-05	1.9074137
**34**	d15_YES	Is English the primary or main language spoken among those living in your household?	7.33E-05	1.217786404
**35**	white_only	Describe your race.	7.29E-05	1.335957195
**36**	qnsc7_MALE	What is your sex or gender?	7.12E-05	1.152331741
**37**	d13_.500.TO..999	What is your total MONTHLY household income, before taxes? Please include income from wages and salaries, remittances from family members living elsewhere, farming, and all other sources.	6.95E-05	1.185767829

**38**	fp4_1_REFUSED	Can you give me a ballpark figure for the amount you have set aside?	6.72E-05	0.928492335
**39**	qnsc7_FEMALE	What is your sex or gender?	6.37E-05	1.089689806
**40**	a1a_orig201_	How did you get the information that you read, saw, or heard about getting better prepared for a disaster?	6.13E-05	1.08664111
